# *In vitro* embryo production in buffaloes: from the laboratory to the farm

**DOI:** 10.21451/1984-3143-AR2018-0135

**Published:** 2019-10-23

**Authors:** Diego Fernando Dubeibe Marin, Eduardo Baia de Souza, Vanessa Cunha de Brito, Carlos Vinicius Nascimento, Anelise Sarges Ramos, Sebastião Tavares Rolim Filho, Nathalia Nogueira da Costa, Marcela da Silva Cordeiro, Simone do Socorro Damasceno Santos, Otavio Mitio Ohashi

**Affiliations:** 1 Instituto de Ciências Biológicas, Universidade Federal do Pará (UFPA), Belém, Pará, Brazil.; 2 BUBRAS, Belém, Pará, Brazil.; 3 Setor de Reprodução Animal, Universidade Federal Rural da Amazônia (UFRA). Belém, Pará, Brazil.; 4 Instituto Federal de Educação, Ciência e Tecnologia do Pará (IFPA), Belém, Pará, Brazil.

**Keywords:** buffalo, breeding programs, *in vitro* embryo production

## Abstract

Transvaginal follicular aspiration technique together with *in vitro* embryo production are the biotechnological alternatives currently available to support genetic improvement breeding programs in buffalo species. However, aspects related to animal management, lack of knowledge of the metabolic needs and biochemical peculiarities of gametes and embryos, as well as the reproductive physiology characteristics have hampered progress in the results. Despite the low availability of good quality oocytes collected after OPU in donors as a physiological characteristic of buffalo species, high rates of oocyte maturation, modest embryo cleavage, blastocyst production and pregnancy rates after transvaginal embryo transfer in recipients could be obtained in buffalo *in vitro* embryo production programs. The results of implementing an *in vitro* embryo production program in buffaloes in the northern region of Pará state, Brazil, and results published by other groups demonstrate the feasibility of implementing this biotechnology in the routine of breeding programs. Nevertheless, in order to achieve better and consistent results, it is necessary to deepen the knowledge on the peculiarities of reproductive biology in this specie. Selection of donor animals based on ovarian size and ovarian follicular reserve and on the rate of blastocyst production is presented as an effective alternative to increase the efficiency of the *in vitro* embryo production technique applied to the buffalo species.

## Introduction

Considering the limited success in embryo recovery rates with multiple ovulation and embryo transfer programs (MOET) in bubaline species (Drost, 2007; Baruselli *et al*., 2013), transvaginal follicular aspiration technique (Ovum Pick Up [OPU]), together with *in vitro* embryo production are the biotechnological alternatives currently available to support breeding programs in this species (Gimenes, 2010; Gasparrini, 2013; Saliba *et al*., 2013; Galli *et al*., 2014; Ferraz *et al*., 2015; Ohashi *et al*., 2017).

Implementation of the OPU technique in buffalo species was first reported in 1994 (Boni *et al*., 1994), and the first buffalo produced using the combination of OPU and *in vitro* fertilization (IVF) techniques was reported by Galli *et al*., 1998. Since that time, different laboratories with scientific and/or commercial character have worked in the search to improve the embryo production rates.

However, some aspects related to animal management, a lack of knowledge on the metabolic needs and biochemical peculiarities of gametes and embryos, as well as the characteristics of their reproductive physiology have hampered progress in the results (Gasparrini, 2007; Ohashi *et al*., 2017).

The present review describes some of the particularities of the buffalo species in relation to the *in vitro* embryo production technique and the strategies which have been adopted over time in the search to improve embryo production rates, the quality of produced blastocysts and the progress in embryo cryopreservation technics. In addition, some results are described as a product of implementing an *in vitro* embryo production program in a commercial buffalo breeding system in the northern region of Pará state, Brazil.

## Cumulus-oocyte complex (COCs) from live donors

In contrast to the bovine zebu breed, females of the buffalo species show a lower ovarian follicular reserve (~19000 vs ~150000, for buffalo and bovine species respectively) (Danell, 1987; Gasparrini, 2002; Santos *et al*., 2013) and a smaller number of follicles recruited in each wave of follicular growth (around 5 - 8) (Baruselli *et al*., 1997). As a consequence, oocyte recovery rates achieved by OPU are usually lower in buffalo (around 14 vs 37 oocytes/animal for buffalo and bovine [zebu breed] species, respectively) (Gimenes, 2010).

Similarly, the rate of good quality oocytes recovered with morphological characteristics of structures with the possibility of being successfully fertilized is lower (~ 9 COCs/animal) than that obtained in zebu bovine species (around 27 COCs/animal) (Gimenes, 2010; Suresh *et al*., 2009). The high incidence of naturally occurring follicular atresia in the ovaries of the buffaloes could be at least partly responsible for this peculiarity (Ocampo *et al*., 1994; Perera, 2011; Santos *et al*., 2013).

In addition, the number of follicles available for each aspiration session and the quality of the recovered structures are characteristics that may be influenced by climatic seasons. It has been observed that the quantity (6.2 ± 1.4 vs 2.7 ± 0.9 oocytes/ovary during winter and summer seasons, respectively) and quality of oocytes decreases significantly during the hottest periods of the year (Abdoon *et al*., 2014).

However, evidence indicates that these are highly variable characteristics among individuals, but at the same time they are quite repeatable attributes within the same animal over several OPU sessions. Therefore, it is possible to predict the potential of each donor, not only in the oocyte production rate per OPU session, but also for its subsequent embryo production through this biotechnology (Gasparrini *et al*., 2014b).

The frequency at which OPU sessions are performed could affect the number of follicles available for aspiration (Ferraz *et al*., 2015). For example, Konrad *et al*. (2017) observed that 14-day intervals between OPU sessions would increase the amount of recovered oocytes (4.5 ± 0.49/animal) compared to 7-day intervals (2.8 ± 0.45/animal). However, in the aspirations performed every 14 days, the availability of good quality oocytes decreases, therefore generally speaking the frequency of aspirations could affect the oocyte recovery rate, but would not modify the final blastocyst production rate (Konrad *et al*., 2017).

Finally, the length of time that donors undergo OPU may negatively affect the quantity, but mainly the quality of the recovered COCs, leading to a significant decrease in the blastocyst production when the OPU sessions are maintained uninterrupted for a long time (Neglia *et al*., 2011).

In our experience, by following a regimen of follicular aspiration sessions every 15 days over a period of five months of work (Table 1), an average of 13.5 ± 5.6 follicles were aspirated from each animal, from which 10.2 ± 6.5 structures were recovered and 5.2 ± 3.9 COCs selected to continue the *in vitro* maturation process, but only 3.1 ± 2.6 COCs/animal/aspiration session were considered viable according to the morphological characteristics of the COCs (Di francesco *et al*., 2012).

**Table 1:** Mean number and its equivalent in proportion of the number of follicles and structures recovered by follicular aspiration guided by ultrasonography/animal/session, in buffaloes from the northern region of the Pará state, Brazil.

	Aspirated follicles	Recovered structures	COCs taken to IVM	Viable COCs
(n)	13.5 ± 5.6	10.2 ± 6.5	5.2 ± 3.9	3.1 ± 2.6
(%)	-	76%	51%	23.4%

Values of number (n) represent mean ± standard deviation. The proportion (%) of COCs taken to IVM and viable COCs was calculated from the total of recovered structures. COCs: cumulus-oocyte complexes; IVM: *in vitro* maturation.

In these conditions, 76% of the structures were retrieved from the total of available follicles to be aspirated. However, only 51% of this total could be used for IVM purposes, and finally only 23.4% of the COCs were considered viable for IVF (oocytes with more than two layers of cumulus cells). These results confirm that one of the main obstacles in implementing this biotechnology in the buffalo species is related to the low availability of good quality oocytes as raw material for the *in vitro* embryo production process (Table 1).

Additionally, the average of COCs recovered and considered as viable/donor/OPU session at the beginning of the project was 2.5. Upon selection which was subsequently performed through gynecological evaluation by trans-rectal palpation and ultrasonography, buffaloes were selected from the herd that had a larger ovarian size and a follicular population above 10 follicles/ovary (Ohashi *et al*., 2017). Thus, a mean of 3.6 viable COCs/donor/OPU session was achieved in the subsequent stages, demonstrating that the repeatability of the variables associated with the amount of aspirable follicles/animal, represents an usable alternative in the search to increase the availability of viable COCs.

## *In vitro* maturation (IVM)

Buffalo oocytes reach high maturation rates (~ 80%) when taken to culture in laboratory conditions, similar to those obtained in bovine species (Santos *et al*., 2002b; Suresh *et al*., 2009; Gimenes, 2010). Traditionally, the medium and culture conditions are the same as those used for bovine oocytes (Galli *et al*., 2003).

The presence of an appropriate layer of cumulus cells along with the addition of antioxidant substances and growth factors have been considered as essential factors to obtain an adequate nuclear and cytoplasmic maturation process in buffalo oocytes (Gasparrini *et al*., 2006; Singhal *et al*., 2009). According to Surresh *et al*. (2009), the best maturation, cleavage and embryo production rates in buffalo species are achieved when oocytes with a proper cumulus cell layer from large follicles and in the cold seasons of the year are cultured in TCM-199 media supplemented with fetal bovine serum (FBS), FSH and cysteamine (Suresh *et al*., 2009).

In turn, the time required for complete nuclear maturation of oocytes in vitro, which means the arrival of the chromosomes to Metaphase II stage, could be from 18 to 24 h (Santos *et al*., 2002a; Gasparrini *et al*., 2008). Particularly, the increase in the maturation period of buffalo oocytes in the laboratory is related to a decrease in the blastocyst production rates (Oba and Camargos, 2011). Maturation periods longer than 24 hours may lead to inappropriate chromatin configurations, oocyte aging and a decrease in competence development, thus demonstrating more sensitivity to the effects of time on oocyte quality than other species (Kumar and Anand, 2012).

## *In vitro* fertilization (IVF)

In general, cleavage rates after the *in vitro* fertilization process of buffalo oocytes are low (~45-50%) (Suresh *et al*., 2009) compared to those obtained in bovine species (~70%) (Sales *et al*., 2015). This step in the embryo production process is highly influenced by the characteristics of the semen used (Galli *et al*., 2001).

At the beginning of applying the *in vitro* embryo production technique in buffaloes, low quality of the frozen/thawed semen was considered one of the main limiting factors for applying the technique in this species (Totey *et al*., 1992). However, improvements in processing and freezing buffalo semen circumvented this difficulty. Still, differences among bulls in sperm survival after the cryopreservation process also reflect differences in embryo production rates under laboratory conditions (Gasparrini, 2002).

The methods for sperm preparation prior to the *in vitro* fertilization process, which include washing and sperm capacitation procedures, are traditionally the same used for embryo production in the bovine species (Parrish, 2014). Heparin has been chosen to be used as the agent for inducing *in vitro* sperm capacitation in most of the described protocols. However, other substances such as nitric oxide donors (Jagan *et al*., 2012), methyl-β-cyclodextrin (Gasparrini *et al*., 2014a), osteopontin (Boccia *et al*., 2013) or progesterone (Boccia *et al*., 2006), have shown promising results as alternatives to heparin for inducing sperm capacitation in buffaloes.

The co-incubation period of oocytes with sperm cells should be adequate to enable correct induction of plasma membrane vesiculation and the complete acrossomal reaction. Appropriate times of not less than 4-6 h have been described, however, it was reported that this time should be extended for at least 16 h in order to maximize blastocyst production rates (Kumar and Anand, 2012).

On the other hand, a prolonged co-cultivation period could hinder the final blastocyst production rate. Cultivation periods greater than 20 h could increase polyspermy incidence, thus reducing the embryo production rates (Gasparrini *et al*., 2008).

A determinant factor for a good result in the *in vitro* fertilization process is related to the quality and competence characteristics of the oocytes, both acquired during the maturation process. Differences in cleavage and production of blastocyst rates produced from oocytes activated by parthenogenesis as compared to laboratory-fertilized oocytes could indicate that the low rates of development during cultivation would be due to deficiencies in the male gamete, rather than the quality of oocytes (Yang *et al*., 2012). However, evidence that cleavage rates could be increased by supplementation with substances with antioxidant capacity during IVM could indicate that the consequences of fertilization in the laboratory not only depend on the quality of sperm, but also on the two gametes involved in the process (Gasparrini *et al*., 2006).

Finally, differences in the sperm concentration used to obtain adequate cleavage rates may be necessary in the *in vitro* embryo production systems of buffaloes compared to those used in the same process applied to the bovine species (Neglia *et al*., 2003).

During our particular experience, conventional frozen/thawed semen from 2 different bulls were used for the *in vitro* fertilization process, and in some cases the result was evaluated with sexed semen. For this step of the procedure, a concentration of 2 million spermatozoa/mL was used in previously matured oocytes for 20h in TCM-199 medium supplemented with gentamicin (50 μg/mL), epidermal growth factor (EGF) (20 μg/mL), β-mercaptoethanol (50 μM), FSH (0.5 μg/mL), LH (50 μg/mL), ITS (10 μL/mL), sodium pyruvate (0.2 mM) and 10% FBS.

Although the cleavage rate was not evaluated, the final blastocyst production assessed over the total of structures put in maturation showed similar percentages between bulls (21.8 ± 4.3% vs 23 ± 3.0%, mean ± s.e.), whereas the result observed for sexed semen was 17 ± 6.5% (mean ± s.e.). Differences in the quality of sperm cells from conventional semen with respect to sexed semen as a result of the changes induced during the sperm selection process could explain these results (Rath *et al*., 2009; Balao da Silva *et al*., 2012).

## *In vitro* culture (IVC)

Different culture approaches have been tested for buffalo embryos. Procedures such as embryo culture in sheep oviducts (Galli *et al*., 1998), somatic cell co-culture systems (Dantas, 2002), use of culture media supplemented with blood sera or semi-defined media supplemented with BSA (Wadhwa *et al*., 2009) have been evaluated in the *in vitro* production of embryos. However, regardless of the system used, blastocyst formation rates are usually lower than those achieved in cattle (around 22% vs 40%, for buffalo and bovine species, respectively) (Cavalieri et al., 2018; Suresh et al., 2009).

The lack of information about metabolic and biochemical needs of buffalo embryos has made it difficult to develop suitable culture media for the species. Moreover, the observed improvements in the blastocyst production rates could mainly be due to changes in the *in vitro* maturation and fertilization systems, rather than changes tested during the culture period (Gasparrini, 2013). Embryos of the buffalo species develop 12-24 h faster than bovine embryos under *in vitro* or *in vivo* conditions, indicating that the metabolism of these structures has particular features (Galli *et al*., 2001).

For instance, regarding carbohydrate metabolism, different from that observed in other ruminant species (bovine and ovine), buffalo embryos require glucose for their proper development from the earliest cultivation stages (Suárez Novoa *et al*., 2011; kumar *et al*., 2012). It has been shown that relatively high glucose concentrations (1.5 mM) are required during the early development of buffalo embryos around day 4 of cultivation, while a decreased concentration or absence of glucose even in the late cultivation stages did not show deleterious effects (Gasparrini, 2013). Supplementation of culture media with high glucose concentrations (5.6 mM) during oocyte maturation and embryo culture showed promising results (Kumar *et al*., 2012).

On the other hand, the substitution of a part of the culture medium during the IVC period as a strategy to renew embryotrophic factors and to remove toxic products derived from the metabolism did not show differences in the blastocyst production rates. Embryos of this specie would be more sensitive to variations in temperature and pH derived from the handling required to perform the medium exchange, so it is convenient in buffalo *in vitro* embryo production systems to not modify the culture conditions until the final cultivation period (Palta and Chauhan, 1998).

In our case, the embryos were cultured for 6 days in SOF medium supplemented with 5% FBS, in an incubator at 38.5°C, 5% CO_2_, 5% O_2_, and 90% N_2_, without carrying out substitutions of culture medium. The mean blastocyst production per donor/OPU session was 1.2 ± 1.4 (Table 2). However, as observed in the standard deviation value, there was high individual variability in embryo production (0-3.5 embryos/animal/OPU), suggesting that the conditions and culture media used adequately fit the needs of some individuals, but may be insufficient to meet the particular circumstances of other animals kept under even the same management conditions.

**Table 2:** Results of implementing an *in vitro* embryo production program in buffaloes in the northern region of Pará state, Brazil.

Result	Total	Average/aspirated donor	%
OPU Sessions	9	-	-
Embryos produced	151	1.2 ± 1.4	-
Embryos/oocytes in IVM	-	-	23.4
Embryos/viable oocytes	-	-	39.7
Transferred embryos	89	0.7 ± 1.2	-
Pregnancy	19	-	22.2 ± 12.1

Values of proportion (%) of embryos produced from oocytes in IVM and from viable oocytes were calculated from the total number of structures and do not represent mean values.

Through selection of the donors made with reference to the average production rate of embryos, it was possible to increase the average embryo production of 0.7/animal/OPU to 1.2 embryos/animal/OPU. Thus, the selecting individuals based on higher than average blastocyst production rates of animals from the same herd could be a valid strategy to increase embryo production rates by *in vitro* fertilization, thereby making the genetic breeding programs in the buffalo species that use *in vitro* embryo production feasible as a tool.

## Cryopreservation of embryos

Cryopreservation is a technique available to support the commercial programs of embryo production in buffalo, since it enables flexibility in embryo transfer schedules by allowing the transfer of embryos to the more favorable seasons of the year, as well as simplifying the transport and marketing of genetic material (Mandawala *et al*., 2016).

The different cryopreservation methods traditionally used in other domestic species, i.e. the slow freezing and vitrification have been tested on oocytes and embryos from buffalo species (Parnpai *et al*., 2016). However, particularities which are mainly associated to the content and composition of the intracytoplasmic lipids of gametes and embryos of this species (Boni *et al*., 1992), have hampered progress in the results.

Comparatively, in terms of cell survival and the subsequent pregnancy rate, vitrification seems to be the most appropriate cryopreservation method for oocytes and buffalo embryos. For embryos, although calves have been obtained through slow freezing, the results of pregnancy rates for embryos derived from *in vitro* fertilization are low (~24%) (Galli *et al*., 2012), whereas it has been possible to obtain good pregnancy rates (~37%) and healthy offspring through vitrification (Saliba *et al*., 2013).

Nevertheless, certain factors could influence the response of the embryos to the cryopreservation process. The embryo development stage, the composition of the vitrification solutions and the equilibration times used, as well as the embryo origin (*in vivo* or *in vitro*), and the morphological quality of the structures have been pointed out as being responsible for the differences observed in the results (Parnpai *et al*., 2016).

Evidence indicates that embryos at an advanced development stage (expanded blastocyst) better support cryopreservation compared to younger embryos (Hufana-Duran *et al*., 2004). The presence of a larger amount of embryonic cells prior to cryopreservation process allows an adequate proportion of blastomers to survive after thawing, thereby ensuring the continuity of embryonic development.

A combination of cryoprotectants with and without ability to penetrate the cytoplasmatic membrane cell are traditionally used in vitrification protocols. Yang *et al*. (2012) tested different combinations and times, observing that the best results were obtained when embryos were produced *in vitro* with 6 to 7 days of development, and were vitrified in a solution composed of 20% ethylene glycol + 20% DMSO + 0.5 M sucrose (Yang *et al*., 2012).

Meanwhile, regarding the effect of sex on survival after cryopreservation, Mahmoud *et al*. (2015) observed that there was a tendency to an increase in post-devitrification resistance for male embryos even without a significant difference, suggesting that the higher development velocity of male embryos compared to their female counterparts gives these embryos more cells and consequently greater ability to withstand the deleterious effects of cryopreservation (Mahmoud *et al*., 2015).

The supplementation of culture media with chemical substances which influence the energy metabolism has been evaluated as a viable alternative to increasing the efficiency of vitrification in mammalian embryos (Held-Hoelker *et al*., 2017). Adding L-carnitine during *in vitro* culture of buffalo embryos showed an increase in post-devitrification survival rates (Verma *et al*., 2018). Mechanisms associated with this positive response are related to their effect of increasing the intracytoplasmic fatty acids oxidation, in addition to its antioxidant capacity (Abdelrazik *et al*., 2009), thereby reducing the intracytoplasmic lipid content and protecting embryonic cells of oxidative stress, respectively.

During the course of our project, no methods for embryo cryopreservation were used due to the need to prioritize calf production. In fact, the main difficulty along the project was related to the unstable pregnancy rate after embryo transfer (Table 2). Factors related to the experience of the technician, to the sanitary control of the recipients, the quality of the embryos, and even the management of the recipients, as well as the age and quantity of calves delivered (recipient buffaloes from 4 to 12 years old vs. heifers and cows with no more than one birth) could have influenced the results.

## Conclusion

Although there are still limitations in the development of *in vitro* embryo production systems in the buffalo species on a commercial scale, publication of papers demonstrating the possibility of obtaining a reasonable blastocyst rate and adequate pregnancy rates after embryo transfer, both fresh as well as cryopreserved, demonstrate the feasibility of implementing this biotechnology in the routine of breeding programs. However, in order to achieve more appropriate and stable results, and thus provide economic viability to the programs, it is necessary to deepen the knowledge on the peculiarities of the reproductive biology of this specie.

Implementation of selection criteria for oocyte donor animals based on anatomical and physiological characteristics such as ovarian size and ovarian follicular reserve, and on the performance of structures collected after fertilization in the laboratory (final rate of blastocyst production), is presented as an effective alternative to increase the efficiency of the *in vitro* embryo production technique applied to the buffalo species.
